# Dietary inclusion of high-amylose cornstarch increased *Lactobacillus* and *Terrisporobacter* and decreased *Streptococcus* in the cecal digesta of weanling pigs

**DOI:** 10.1093/jas/skaf008

**Published:** 2025-01-18

**Authors:** Michael O Wellington, Seidu Adams, Jung W Lee, Atta K Agyekum, Tofuko A Woyengo

**Affiliations:** Swine Research Centre, Trouw Nutrition R&D, Boxmeer, The Netherlands; Department of Animal Science, University of Nebraska-Lincoln, Lincoln, NE 68588, USA; Department of Veterinary and Biomedical Sciences, South Dakota State University, Brookings, SD 57007, USA; Department of Animal Science, South Dakota State University, Brookings, SD 57007, USA; Trouw Nutrition Canada, Puslinch, ON N0B 2J0, Canada; Department of Animal Science, South Dakota State University, Brookings, SD 57007, USA; Department of Animal and Veterinary Sciences, Aarhus University, Tjele, Denmark

**Keywords:** canola cake, cornstarch, *Lactobacillus*, microbiome, piglets, volatile fatty acids

## Abstract

The study investigated the effect of dietary inclusion of high-amylose cornstarch (**HA-starch**) on cecal microbiota composition and volatile fatty acid (**VFA**) concentrations in weanling pigs fed high levels of cold-pressed canola-cake (**CPCC**). Weaned pigs (240 mixed sex; 7.1 ± 1.2 kg) were housed in 40 pens (6 pigs/pen) and fed a common commercial diet for 7 d, followed by the experimental diets for 28-d, which contained either 0% or 40% CPCC with either 0% or 40% HA-starch. At the end of the study, one pig from each pen (*n* = 8) was selected and euthanized to collect cecal digesta for microbial and VFA composition analyses. The HA-starch increased (*P* < 0.001) acetate, propionate, and butyrate concentrations, thereby increasing total VFA concentration (*P* < 0.001). There was a tendency for cecal butyrate and total VFA concentrations to decrease when pigs were fed the 40% CPCC diet without HA-starch but increase when fed the 40% CPCC diet containing 40% HA-starch (CPCC × HA-starch effect; *P* = 0.09), indicating HA-starch can increase cecal butyrate and total VFA concentrations in pigs fed a diet with high-CPCC level. The proportions of *Lactobacillus* and *Terrisporobacter* were high, whereas low proportions of *Streptococcus* genus were observed in the cecal microbiota of pigs fed diets containing 40% HA-starch. Also, pathways consistent with carbohydrate digestion, absorption, and phosphate metabolism were enriched in pigs when the diet included 40% HA-starch. In summary, incorporating high amounts of HA-starch in a weanling pig diet containing high levels of CPCC may benefit intestinal health and digestive performance by enhancing the abundance of probiotic commensal bacteria, contributing to increased enzymatic activity and carbohydrate metabolism.

## Teaser

High-amylose cornstarch could be a valuable dietary addition to support the intestinal health and overall well-being of weanling pigs, especially when using canola meal as a protein source.

## Introduction

Weaning is a critical period in a piglet’s life, during which the transition from the sow’s milk to a solid diet causes many changes in intestinal development, digestive capacity, and immune system regulation. Therefore, providing the piglets with adequate nutrition is essential to support intestinal development and optimal growth. Incorporating prebiotics (fermentable fiber sources) in pre- and postweaning piglet diets supports intestinal development, health, microbiome composition, and abundance, leading to improved growth performance (Hong et al., 2020, 2021a). A recent study by Hong et al. (2021a) observed that the inclusion of sugar beet pulp in the diet of weaned piglets increased feed efficiency and abundance of intestinal health-promoting bacteria *Butyricicoccus* in the colon. Dietary inclusion of canola meal in weaning piglets’ diet has improved growth performance, feed efficiency, and nutrient digestibility (Landero et al., 2011; Hong et al., 2021a, 2021b). In addition, including 20% canola meal in a corn–soybean meal-based diet increased the growth performance, microbial composition, and immune response in nursery piglets (Hong et al., 2020). Canola co-products such as cold-pressed canola cake (**CPCC**) have high insoluble fiber content that can reduce *E. coli* infections in weaned pigs by increasing the digesta passage rate in the small intestine (Hong et al., 2020, 2021a). High inclusion (40%) of CPCC has been reported to negatively impact growth performance, by reducing thyroid and liver function (Lee and Woyengo, 2018). However, at low inclusion levels, the canola co-products such as CPCC contain glucosinolates that have antioxidant and antimicrobial activity against pathogenic microorganisms in the intestine (Mejicanos et al., 2017; Hong et al., 2020). It is thought that the inclusion of HA-starch can act as a substrate of fermentation and mitigate the negative effects of the toxic metabolites from glucosinolates. This is because, the amino acid digestibility of canola co-products, especially CPCC, is lower than that of conventional feedstuffs such as soybean meal (**SBM**) and corn, resulting in an increased flow of undigested amino acids into the large intestine (Grageola et al., 2013; Berrocoso et al., 2015). The presence of these undigested proteins in the large intestine stimulates the growth of proteolytic microorganisms that ferment proteins into various toxic metabolites such as ammonia, amines, indoles, and phenols that negatively affect the intestinal health of the piglets (Pieper et al., 2012; Richter et al., 2014; Zhang et al., 2020).

High-amylose cornstarch (**HA-starch**) has a high starch content that escapes digestion in the small intestine (resistant starch). This resistant starch can promote the growth of amylolytic microorganisms at the expense of proteolytic microorganisms, leading to a shift in the fermentation of undigested proteins from the production of toxic metabolites such as ammonia towards the production of microbial protein, which is excreted via feces (Birt et al., 2013). Also, the toxicity of glucosinolate degradation products is partly dependent on the pH of the fermentation medium (digesta); the toxicity reduces with a decrease in the pH of the digesta (Barba et al., 2016; Abdel-Massih et al., 2023). Thus, resistant starch in the HA-starch can reduce the toxicity of glucosinolate degradation products by decreasing the pH of large intestine digesta through increased production of volatile fatty acids (**VFA; **Lee et al., 2020). Indeed, in a previous study (Lee et al., 2020), the authors demonstrated that including HA-starch in CPCC-containing diets reduced the production of more toxic glucosinolate degradation metabolites in the cecum of weaned pigs. The change in the gastrointestinal pH, composition of glucosinolate degradation products, and toxicity of glucosinolates due to dietary inclusion of resistant starch implies a change in gastrointestinal microbial composition and volatile fatty acid production due to the dietary inclusion of the resistant starch. Including HA-starch in the diet of weaning piglets was reported to improve nutrient digestibility, intestinal health, and growth performance (Fouhse et al., 2015; Gao et al., 2020; Park et al., 2020). However, information is lacking on the effects of dietary inclusion of CPCC in combination with HA-starch on the composition of large intestinal microorganisms and VFA and branched-chained fatty acid (**BCFA**) concentration in weaned pigs.

We hypothesized that including HA-starch in a weanling pig diet containing cold-pressed canola-cake will result in cecal microbiota composition and VFA concentration that benefit intestinal health. Our objective was to determine the independent or interactive effect of adding **CPCC** (0% or 40% dietary inclusion) and **HA-starch** (0% or 40% inclusion) to the diet of weanling pigs on VFA and BCFA concentration in the cecum and subsequent changes in the microbiome composition and abundance.

## Methods

The Institutional Animal Care and Use Committee reviewed and approved the experimental animal procedures (South Dakota State University #18-076E). All procedures were done according to the stipulated animal care and handling guidelines at South Dakota State University.

### Animals housing, diets, and sampling

The experimental setup and diet details have been previously published (Lee et al., 2020). Briefly, 240 mixed-sex pigs (7.1 ± 1.2 kg) weaned at 21 d were fed a common commercial starter diet during the first 7-d postweaning, followed by the experimental diets for 28 d. The experimental diets consisted of a basal diet that contained either 0% or 40% CPCC with either 0% or 40% HA-starch in 2 × 2 factorial arrangements. The 40% CPCC diet without the HA-starch contained 13.7% NDF, 8.8% ADF, and 22.7% CP. While the 40% CPCC diet with the HA-starch contained 10.5% NDF, 7.7% ADF, and 18.4% CP as previously reported (Lee et al., 2020). The experimental diets were fed as mash and in 2 phases: Phase 1 for the first 14 d, and Phase 2 for the last 14 d of feeding the experimental diet. On the last day of the experimental period, one pig from each pen (*n* = 8 pigs/treatment) with body weight (**BW**) close to the pen average BW was selected and then euthanized. Cecal digesta were collected from the euthanized pigs directly by emptying the cecum, immediately snap-frozen in liquid nitrogen, and later stored at −80 °C for microbial composition analysis and VFA concentration in the cecal digesta.

### Sample preparation and analytical procedures

Cecal digesta samples were thawed and centrifuged at 3,000 × *g* for 30 min at 4 °C. The supernatant was collected for VFA and BCFA analysis, as described by Erwin et al. (1961). Briefly, 0.8 mL of the supernatant was mixed with 0.2 mL of 25% phosphoric acid and 0.2 mL of internal standard solution (150 mg of 4-methyl-valeric acid, S381810; Sigma-Aldrich Corp., St. Louis, MO, USA) and vortexed for 1 min. The concentration of acetic acid, propionic acid, butyric acid, acids branched chain fatty acids (BCFA), and total VFA in the supernatant was determined by gas chromatography (Trace 1310, Thermo Fischer Scientific, Waltham, MA, USA) with a Stabilwax-DA column (30-m × 0.25-mm i.d.; Restek, Bellefonte, PA, USA). A flame-ionization detector was used with an injector temperature of 170 °C. To analyze the microbial composition of cecal digesta, total microbial DNA was extracted from the cecal digesta and fecal samples using the Powersoil® DNA Isolation Kit (QIAGEN, MD) according to the manufacturer’s instructions. The quality of the DNA was determined using NanoDrop one (Thermo Fisher Scientific, DE) and quantified using Qubit Fluorometer 3.0 (Invitrogen, CA). The hypervariable V3-V4 regions of the bacterial 16S rRNA were amplified, and 0.3 ng of DNA was used for library preparation according to the Nextera XT library preparation kit (Illumina, San Diego, CA) and sequenced on the MiSeq Platform. The variations in bacterial communities within the cecal digesta using 16S rRNA microbial community analysis package in Quantitative Insights into Microbial Ecology framework (QIIME, Version 2.0) (Bolyen et al., 2019). Briefly, the samples were demultiplexed, quality-filtered, and denoised using DADA2 (Callahan et al., 2016). The reads were quality filtered, and amplicon sequence variants (**ASVs**) were predicted and assigned to taxonomy using the DADA2-formated trained SILVA database version 138 release (Pruesse et al., 2007). The mothur package (version 1.35.1) was used to calculate the phylogenetic distances between the predicted ASVs and presented in a phylogenetic tree format (Schloss et al., 2011). Differential abundance analysis was performed using the DESeq2 package in R programming (Love et al., 2014).

### Statistical analysis

All data were verified for normality by the Proc Univariate of SAS, and outliers were identified at 3 standard deviations from the mean. The VFA and BCFA data analysis were analyzed as a 2 × 2 factorial with the fixed effect factors of CPCC (0% or 40%; *n* = *2*), HA-starch (0% or 40%; *n* = *2*), and their interactions. The microbiome compositional analysis was performed using the phyloseq (McMurdie and Holmes, 2013) and the microeco package (Liu et al., 2021) in R programming. The permutational multivariate analysis of variance (PERMANOVA) was performed using Vegan version 2.4-2. using the Kruskal–Wallis Sum and Wilcox tests to estimate genus-level microbial community differences (Anderson, 2001) Statistical significance in all analyses was determined at *P* < 0.05.

## Results

### Volatile fatty acids (VFA), branched-chained fatty acid (BCFA), and metabolic pathways in the cecum.

**Figure 1.**
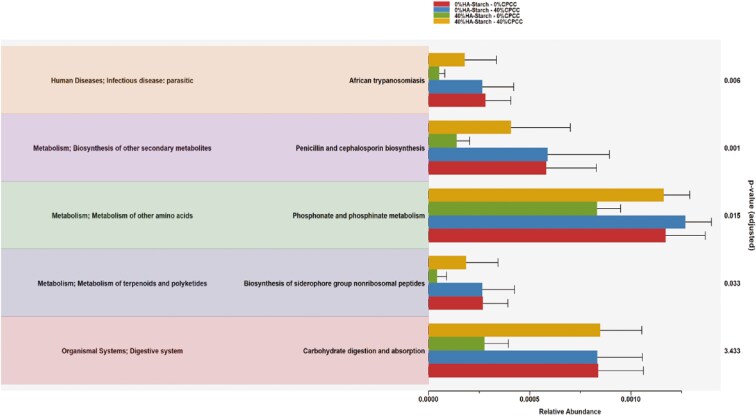
Metabolic pathway of cecal microbiome of piglets. Colors on the left side of the image illustrates the different metabolic pathways and corresponding functions in the cecum of the piglets. The barplots and the different colors to the right side illustrates the different treatment groups. The height of the bars illustrates the proportions of each functions/metabolic pathways.

The VFA concentration data are presented in Table 1. We observed that the inclusion of 40% HA-starch, regardless of the CPCC concentration, significantly increased (*P* < 0.001) the acetate, propionate, and butyrate concentration in the cecal digesta. Consequently, the total VFA concentration was higher (*P* < 0.001) in the 40% HA-starch-based diet regardless of the CPCC concentration. We also observed a tendency for a higher BCFA concentration in the 40% HA-starch-fed piglets (*P* = 0.087), regardless of CPCC concentration. There was also a tendency for a CPCC × HA-starch effect on butyrate (*P* = 0.093) and total VFA (*P* = 0.089). Specifically, cecal butyrate concentration was lowest with the 0% HA-starch + 40% CPCC diet compared to the 40% HA-starch + 40% CPCC diet, indicating HA-starch increased butyrate concentration in the diet containing 40% CPCC. Total VFA tended to decrease with the 0% HA-starch + 40% CPCC but increased with the 40% HA-starch + 40% CPCC-based diet. As presented in Fig. 1, Carbohydrate digestion and absorption activities were reportedly decreased in pigs that received 40% HA-starch diets compared to the other treatment groups. Similarly, we observed that phosphonate and phosphinate metabolism decreased in pigs fed 40% HA-starch diets compared to the other groups.

**Table 1:** **Cecal VFA concentrations of piglets-fed CPCC without or with HA-starch** ^1^.

	HA^2^-starch 0%		HA-starch 40%		SEM	*P*-value		
	CPCC 0%	CPCC 40%	CPCC 0%	CPCC 40%		*CPCC*	*HA*	CPCC*HA
Cecal VFA^3^, m*M* of digesta								
Total VFA	78.6	58.2	108.1	116.3	8.82	0.455	<0.001	0.089
Acetate	44.8	37.0	55.8	61.0	5.07	0.782	<0.001	0.176
Propionate	22.7	16.2	35.4	39.3	4.84	0.779	<0.001	0.248
Butyrate	10.3	4.36	16.0	15.0	1.55	0.022	<0.001	0.093
BCFA^4^	0.85	0.64	0.97	1.01	0.16	0.55	0.087	0.382

^a,b,c^Within a row, means without a common superscript differ (*P* < 0.05).

^1^Pigs were housed in 40 pens (6 pigs/pen), and 1 pig from each pen (8 pigs/diet) with BW close to the pen average BW were euthanized to collect cecal digesta to determine VFA concentrations.

^2^HA-starch = high-amylose cornstarch. HA-starch (Hylon VIITM; 70% amylose) was obtained from Ingredion Incorporated (Westchester, IL).

^3^VFA = volatile fatty acids.

^4^BCFA = branched-chain fatty acids.

### ASVs prediction and microbial community composition

**Figure 2.**
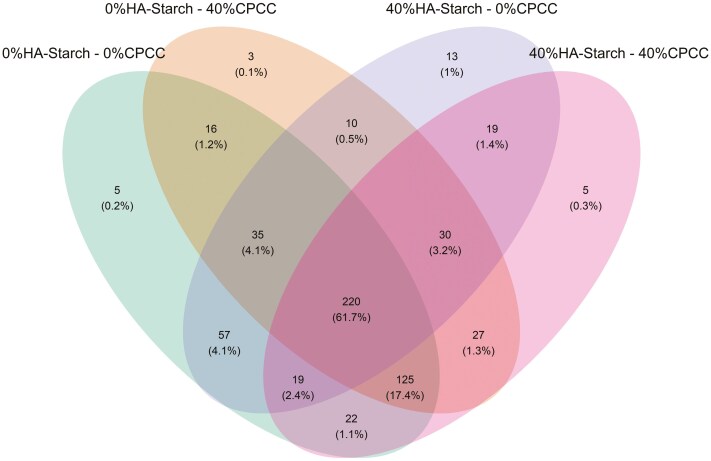
Amplicon sequence variants as distributed between the treatment groups

**Figure 3.**
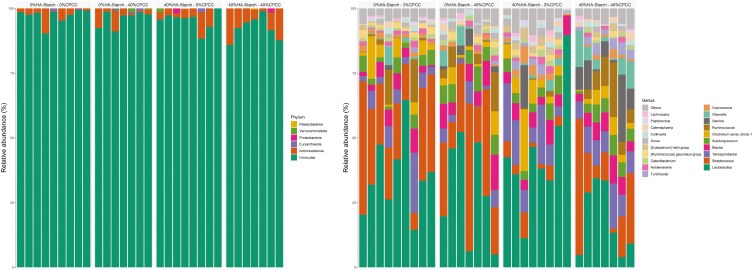
(A). Relative abundance (%) distribution of bacterial communities is illustrated in a stacked bar plot across the different treatment groups. Colors illustrate the different bacterial phyla in each treatment group. (B). Genus-level relative abundance distribution of bacterial communities using a stacked barplot across different treatment groups. With different colors denoting different genera across different treatment groups

A total of 1,489 ASVs were predicted and distributed across all samples within the 4 treatment groups. The 1,489 ASVs were filtered based on abundance (>0.15%) and presence in greater than 2 samples (>2). We retained 606 high-quality ASVs, representing 88.36% of the total reads. To determine the distribution of the high-quality ASVs amongst the 4 treatment groups, we used the trans-Venn class from the microeco package to estimate the shared and unique ASVs across the treatment groups (Fig. 2). About 0.2% (5 ASVs), 0.1% (3 ASVs), 1% (13 ASVs), and 0.3% (5 ASVs) were unique to 0%HA-starch + 0%CPCC, 0%HA-starch + 40%CPCC, 40%HA-starch + 0%CPCC, and 40%HA-starch + 40%CPCC respectively. However, about 61.7% (220 ASVs) were shared amongst all 4 treatment groups, and about 4.1% (57 ASVs) were shared between 0%HA-starch + 0%CPCC and 40%HA + 0%CPCC. 1.4% (19ASVs) were shared between 40%HA-starch + 0%CPCC and 40%HA-starch + 40%CPCC, whereas 1.2% (16 ASVs) were shared between 0%HA-starch + 0%CPCC and 0%HA-starch + 40%CPCC. The most abundant phyla were the *Firmicutes*, *Actinobacteriota*, *Proteobacteria*, *Euryarchaeota*, *Verrucomicrobiota*, and *Patescibacteria* (Fig. 3). The *Firmicutes* phylum was highly abundant with a percentage abundance of 97.19%, 94.65%, 96.10%, and 92.43% in 0%HA-starch + 0%CPCC, 40%HA-starch + 0%CPCC, 0%HA-starch + 40%CPCC, and 40%HA + 40%CPCC, respectively. The percentage abundance of the *Actinobacteriota* phylum was 7.15% (40%HA-starch + 40%CPCC), 4.48% (40%HA-starch + 0%CPCC), 3.30% (0%HA-starch + 40%CPCC), and 2.61% (0%HA-starch + 0%CPCC). At the genus level, the most abundant genera were *Lactobacillus*, *Streptococcus*, *Terrisporobacter*, *Blautia*, *Subdoligranulum*, *Clostridium sensu stricto 1*, *Ruminococcus*, *Sarcina*, *Olsenella, Coprococcus, Turicibacter, Holdemanella, Catenibacterium*, [*Eubacterium*] *hallii* group, and [*Ruminococcus*] *gauvreauii* group.

**Figure 4.**
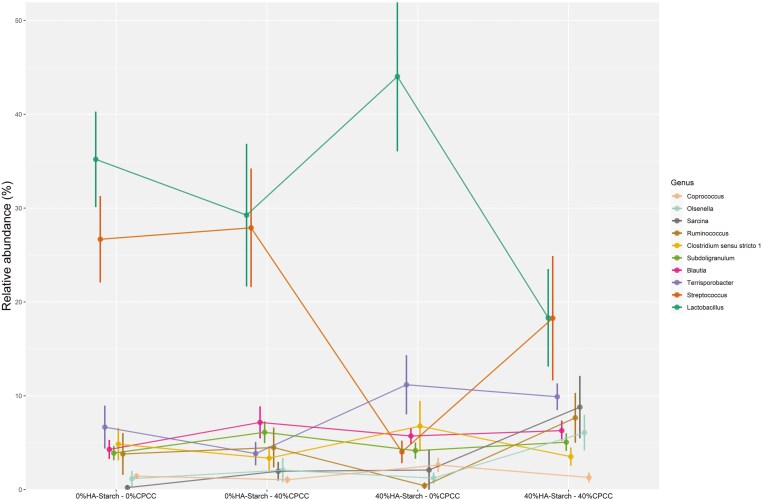
Relative abundance based distribution of different bacteria groups at the genus level across different treatment groups

The addition of HA-starch increased the proportion of *Lactobacillus* and *Terrisporobacter* and decreased the relative abundance of *Streptococcus* (Fig. 4). The proportion of *Lactobacillus* was 41.66% in 40%HA-starch + 0%CPCC, 35.34% in 0%HA-starch + 0%CPCC, 28% in 0%HA-starch + 40%CPCC, and 18.30% in 40%HA-starch + 40%CPCC. Low proportions of the *Streptococcus genus* were observed to be associated with the supplementation of the HA-starch diet: 4.11% in 40%HA-starch + 0%CPCC and 17.87 for 40%HA-starch + 40%CPCC compared to 29.69% in 0%HA-starch + 40%CPCC and 26.75% for 0%HA-starch + 0%CPCC. Interestingly, a high abundance of *Terrisporobacter* was also observed to be associated with dietary supplementation of HA-starch, with 11.85% in 40%HA-starch + 0%CPCC and 9.91% in 40%HA-starch + 40%CPCC compared to 6.44% for 0%HA-starch + 40%CPCC and 3.91% in 0%HA-starch + 0%CPCC.

### Microbiome diversities in the cecal bacterial communities

**Figure 5.**
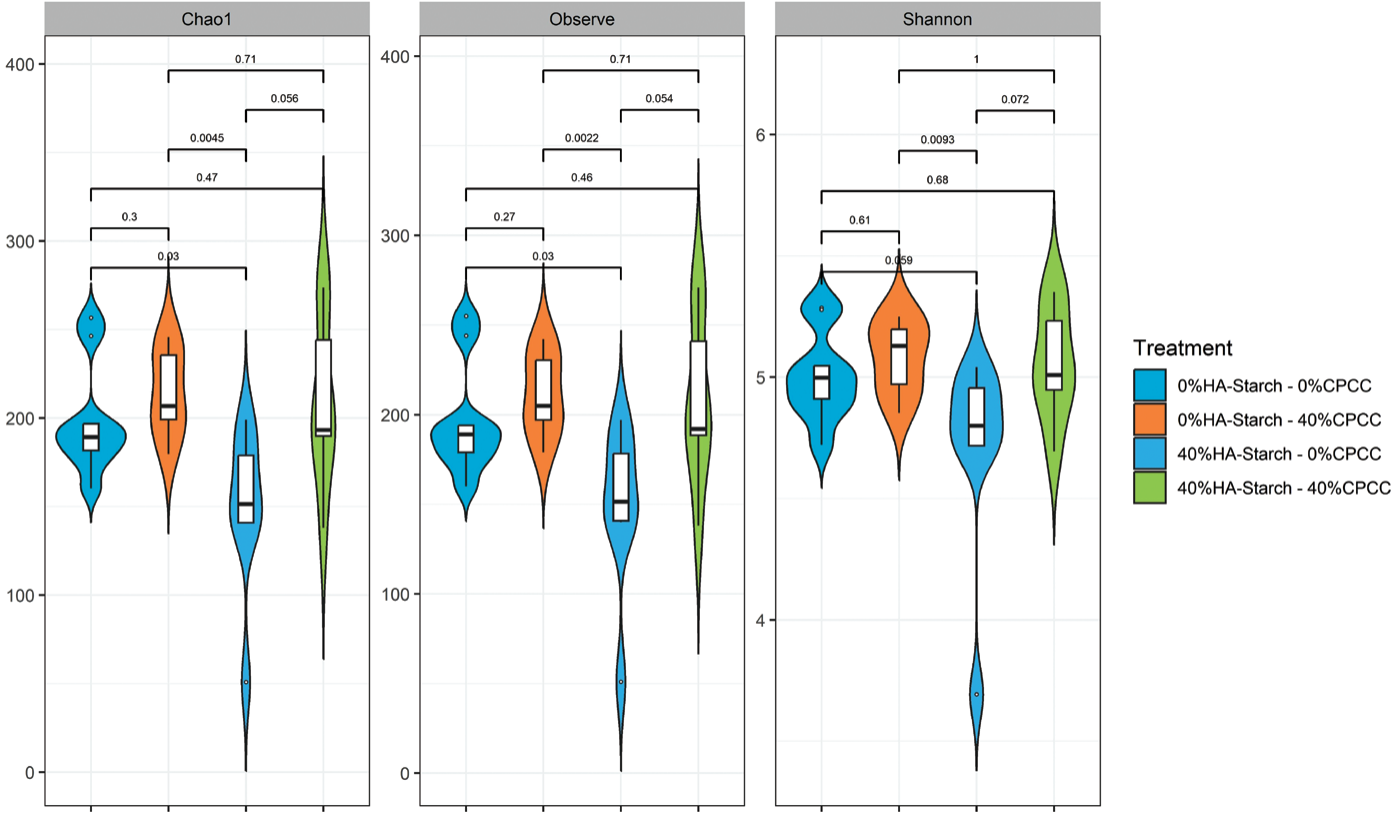
Alpha diversity analysis of cecal bacterial communities across the different treatment groups using the Chao1, Observe, and Shannon indices. Different colors represents the different treatments.

The alpha diversity indices and beta diversity metrics estimated the cecal microbial community diversities. The Chao1, Observed, and Shannon indices were used to determine cecal bacterial community diversities within the treatment groups. As shown in Fig. 5 and Supplementary Fig. 1, the Shannon diversity index, Observe ASVs, and the Chao1 indices showed a significant difference (*P* < 0.05) between 0%HA-starch + 40%CPCC and 40%HA-starch + 0%CPCC.

**Figure 6.**
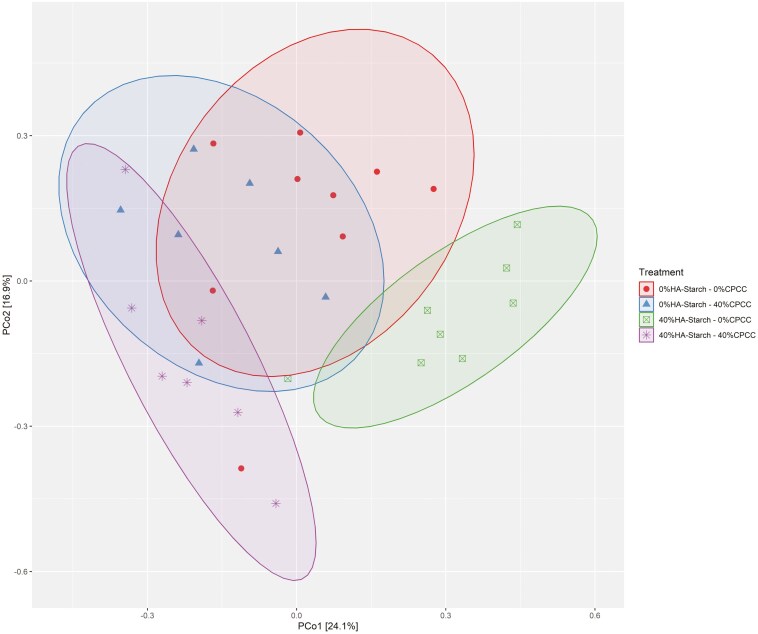
Beta diversity analysis using the Principal coordinate analysis (PCoA) of the cecal bacterial community composition in the different treatment groups. Different colors and eclipse represents the different treatments.

The Observed index showed a significant difference (*P *= 0.0022) between 40%HA-starch + 0%CPCC and 0%HA-starch + 40%CPCC. Similarly, there was a significant difference (*P* = 0.03) between 40%HA-starch + 0%CPCC, 0%HA-starch + 40%CPCC, and 0%HA-starch + 0%CPCC. The Chao1 index showed a significant difference (*P* = 0.0045) between 0%HA-starch + 40%CPCC and 40%HA-starch + 0%CPCC. Shannon diversity was significantly different (*P* = 0.0045) between 0%HA-starch + 40%CPCC and 40%HA-starch + 0%CPCC. The Bray–Curtis distance metric was used to calculate the principal coordinate analysis (PCoA) between the treatment groups, as shown in Fig. 6. We observed a significant difference between the treatment groups (*P *= 0.001, *R*^*2*^* *= 0.2706) with a distinct clustering of 40%HA-starch + 0%CPCC. We estimated the permutation test for homogeneity of multivariate dispersions (PERMDISP) (Anderson et al., 2011) to *P* = 0.887 using the number of permutations 999.

### Differential abundance analysis of cecal bacterial communities

The differential abundance analysis was conducted using the DESeq2 analysis pipeline, employing a log2foldchange cutoff of 0.05. This criterion was applied to identify bacteria that exhibited differential abundance in cecal bacterial communities among various treatment groups.

**Figure 7.**
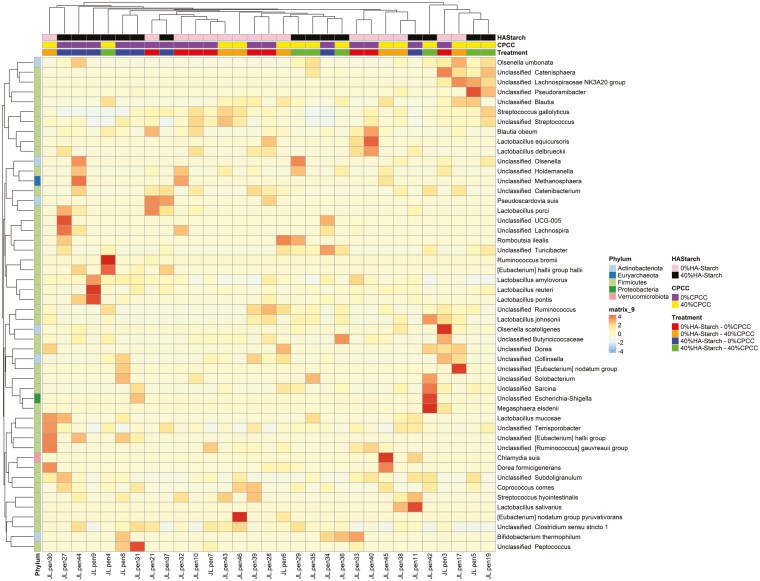
Species level differential abundance (DA) analysis of the different bacterial communities in the piglets cecal samples across different treatment groups.

As illustrated in Fig. 7, about 36 ASVs affiliated with the Lactobacillus genus were differentially abundant in piglets subjected to a diet comprising 40%HA-starch + 40%CPCC. In contrast, approximately 83 bacterial ASVs, spanning the genera Streptococcus, Lactobacillus, Blautia, Subdoligranulum, Ruminococcus, Olsenella, and Butyricicoccaceae, demonstrated differential abundance in piglets fed a diet of 40%HA-starch + 0%CPCC. Five bacterial ASVs associated with the genera Streptococcus, Lactobacillus, Eubacterium, and Catenisphaera exhibited differential abundance in piglets consuming a diet featuring 0%HA-starch + 40%CPCC. In comparison, around 90 ASVs representing various bacterial genera displayed differential abundance in piglets fed a diet of 40%HA-starch + 0%CPCC.

### The abundance of Lactobacillus and Terrisporobacter genera within the treatment groups

**Figure 8.**
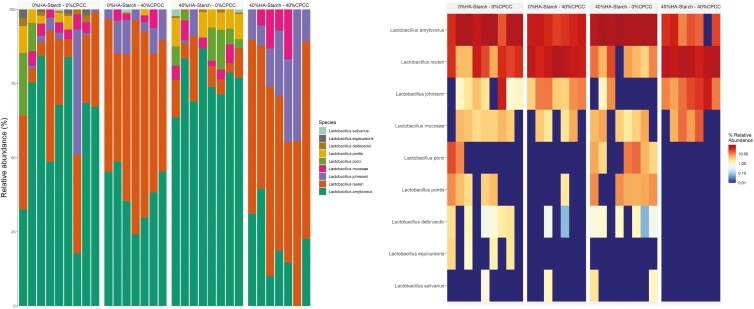
(A). A stacked bar plot of the relative abundance (%) distribution of the different *Lactobacillus species* in the different treatment groups. (B). Relative abundance (%) based heatmap of the different *Lactobacillus species* distribution in the different treatment groups.

**Figure 9.**
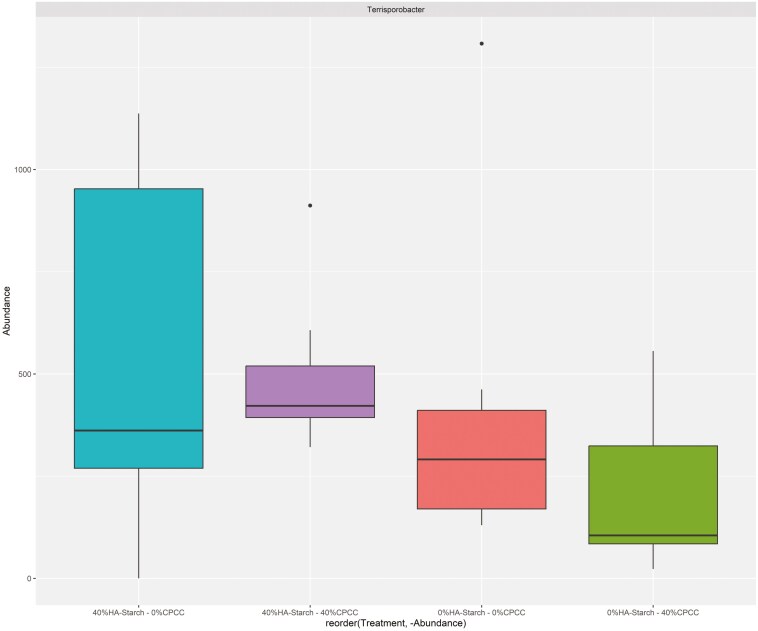
A box plot comparing the relative abundance of the Terrisporobacter species in the different treatment groups

This investigation identified a notable prevalence of the Lactobacillus genus within the treatment groups. Consequently, to ascertain the distribution of Lactobacillus across distinct treatment groups and to delineate the prevalence of different Lactobacillus species within each group, we conducted a comprehensive profiling of the total Lactobacillus reads proportionally across all samples and treatments (Fig. 8). The total reads affiliated with the Lactobacillus genus was observed as follows: 15,044 reads for 40%HA-starch + 0%CPCC, 6,424 reads for 40%HA-starch + 40%CPCC, 18,809 reads for 0%HA-starch + 0%CPCC, and 10,751 reads for 0%HA-starch + 40%CPCC. Our analysis revealed 9 distinct taxa of Lactobacillus species present across the various treatment groups, including *Lactobacillus amylovorus, Lactobacillus delbrueckii, Lactobacillus equicursoris, Lactobacillus johnsonii, Lactobacillus mucosae, Lactobacillus pontis, Lactobacillus porci, Lactobacillus reuteri*, and *Lactobacillus salivarius* (Fig. 8 and Supplementary Fig. 2). Specifically, the abundance of *Lactobacillus amylovorus* across treatment groups was noted as 11,492 in 40%HA-starch + 0%CPCC, 1,410 in 40%HA-starch + 40%CPCC, 12,876 in 0%HA-starch + 0%CPCC, and 4,009 in 0%HA-starch + 40%CPCC. The total reads for *Lactobacillus delbrueckii* were 104, 0, 176, and 16 in 40%HA-starch + 0%CPCC, 40%HA-starch + 40%CPCC, 0%HA-starch + 0%CPCC, and 0%HA-starch + 40%CPCC, respectively. *Lactobacillus equicursoris* was exclusively observed in 0%HA-starch + 0%CPCC, with a total read count of 160. Notably, *Lactobacillus porci* exhibited higher representation in 40%HA-starch + 0%CPCC. Furthermore, incorporating HA-starch in the pig diet was associated with elevated Terrisporobacter. The lowest abundance of Terrisporobacter was observed in pigs fed a diet containing high-CPCC (Fig. 9).

### The abundance of *Streptococcus genera within the treatment groups*

**Figure 10.**
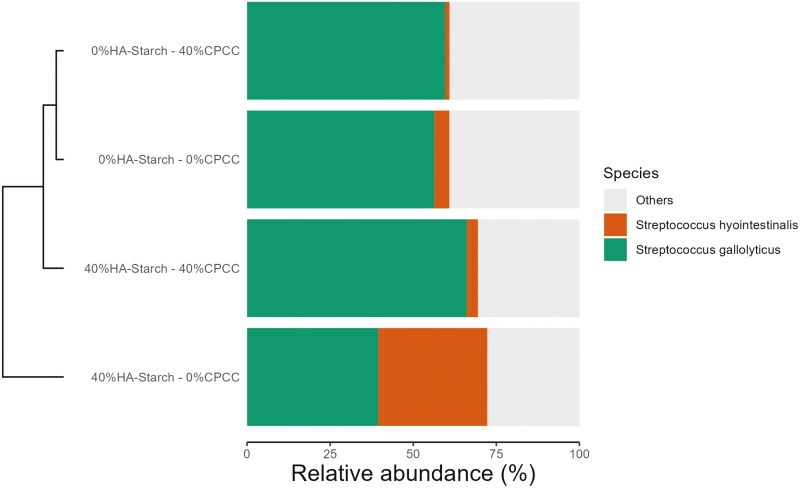
The relative abundance based comparison of the different *Streptococcus* species in the different treatment groups. The *S. hyointestinalis* and *S. gallolyticus* species were highly abundant in the treatment groups relative to other species.

The relative abundance of *Streptococcus* genera was observed to vary within the treatment groups. We observed that the addition of HA-starch decreased the abundance of *Streptococcus* genera; 40%HA-starch + 0%CPCC (4.97%) and 40%HA-starch + 40%CPCC (19.98%) whereas the highest relative abundance of *Streptococcus* was observed in 0%HA-starch + 40%CPCC (28%) (Fig. 4). The most relatively abundant *Streptococcus* species were the *Streptococcus hyointestinalis* and *Streptococcus gallolyticus*. The highest relative abundance of *S. gallolyticus* was observed in 40%HA-starch + 40%CPCC, and the lowest relative abundance was observed in the 40%HA-starch + 0%CPCC. Meanwhile, the highest relative abundance of *S. hyointestinalis* was observed in the 40%HA-starch + 0%CPCC fed pigs, and the lowest relative abundance of *S. hyointestinalis* was recorded in 0%HA-starch + 40%CPCC (Fig. 10).

## Discussion

The present study aimed to investigate whether CPCC and HA-starch inclusion in the diet of weaned piglets will impact VFA concentration in the cecum and cause changes in the microbiome composition and abundance. We have previously reported that including CPCC independently reduced growth performance, increased liver size, and interfered with thyroid gland function, but these were mitigated with the inclusion of HA-starch (Lee et al., 2020). Previous reports indicate that including HA-starch in the diet of weaning piglets improved nutrient digestibility, intestinal health, and growth performance (Lu et al., 2016). Based on the fermentable characteristics of the HA-starch used in the present study, coupled with the relatively high crude protein content of the CPCC, a combined inclusion of both ingredients may improve intestinal health and confer growth performance benefits, because at high-CPCC inclusion we previously reported a reduction in growth performance (Lee and Woyengo, 2018). The present study evaluated the impact of the dietary treatments on VFA concentration in cecal digesta, which showed a consistent increase in acetate, propionate, and butyrate concentration in pigs fed the 40% HA-starch diet. Previous studies have reported similar effects of HA-starch increasing the VFA concentration in the intestinal digesta, especially butyrate concentrations (Demigne et al., 1989; Lu et al., 2016; Kerr et al., 2023). Increased VFA production promotes intestinal development and health, especially for butyrate, improving intestinal epithelial cell function and as an energy source for the cells (Tsukahara et al., 2003; Gao et al., 2020; Salvi and Cowles, 2021; Ma et al., 2022). The production of these VFAs is based on the metabolic outcomes of resident microbiomes in the gut; as such, microbial metabolic pathways are linked with different biochemical activities within the cells that may activate an enzymatic reaction leading to the synthesis of metabolites.

Furthermore, the present study used the PICRUSt2 (Douglas et al., 2020) and ggpicrust2 (Yang et al., 2023) to functionally predict the organic compounds degraded by the bacteria communities within the cecal microbiome of the fed diets with or without HA-starch and CPCC inclusion. Overall, pathways responsible for carbohydrate digestion, absorption, and phosphate and phosphinate metabolism were highly significant and enriched in pigs fed the 40% HA-starch diet and the combined treatment of HA-starch and CPCC. The enriched metabolic pathways indicate an interaction between the host pig and the microbial communities, synthesizing metabolites that have implications for the host’s immunity, metabolism, and physiology, usually with positive outcomes (Visconti et al., 2019; Cox et al., 2022). Since the present study only observed a higher enrichment in the HA-starch-fed pigs, this may indicate the impact of the 40%HA-starch on microbial metabolism, further enhancing the host physiology and health. Bacterial community composition was determined to assess the possible effect of the basal diet with or without CPCC and HA-starch on different intestinal microbial communities of piglets. Indeed, substrate availability and the preference of the microbes to utilize a particular substrate offer a significant factor in determining microbial composition.

In the present study, *Firmicutes* were the most abundant phyla across the treatments. Specifically, the combined effect of HA-starch and CPCC and the individual supplementation of 40%HA-starch decreased the relative abundance of the *Firmicutes* phyla compared to the control or the 40%CPCC diets. *Firmicutes* are usually the most abundant phyla because many of the members of this phylum can break down carbohydrates that escape enzymatic digestion, usually in the form of dietary fiber or resistant starch (Regmi et al., 2011). This is in accordance with our observation that the proportion of carbohydrate digestion and absorption enzymes was relatively lower in the 40%HA-starch treated pigs. There is evidence that *Firmicutes* are efficient producers of butyrate (Macfarlane and Macfarlane, 2003). *Firmicutes* are the most abundant bacteria inhabiting the gut and play a role in the metabolism of energy from the host, which may lead to increased fat accumulation in pigs, as proposed by others (Komaroff, 2017; Lu et al., 2021), but this was not evaluated in the present work. Interestingly, the proportion of *Actinobacteriota* was abundant in the combined treatment of 40% HA-starch and 40%CPCC group and the individual treatment group of 40%HA-starch. This observation contradicts previous studies on the intestinal microbiome, indicating that the *Firmicutes* and *Bacteroidetes* are the most abundant (Ivarsson et al., 2014; Xiao et al., 2017; Crespo-Piazuelo et al., 2018). However, the observation in the present study agrees with Yang et al. (2020), who reported that *Firmicutes* and *Actinobacteriota* were the first and second most abundant phyla in wild pigs.

At the genus level, *Lactobacillus* was the most abundant across the treatments in relation to the other genus. We observed that piglets fed the 40% HA-starch diet had a high abundance of *Lactobacillus* (41.66%), which is consistent with previous reports that have indicated that diets containing HA-starch can increase digesta mass and commensal bacteria population (*Bifidobacterium* spp. and *Lactobacillus*) (Bird et al., 2007; Regmi et al., 2011; Yu et al., 2019). Indeed, *Lactobacillus* is highly amylolytic and can inhibit the colonization of non-amylolytic bacteria, which allows for their colonization, hence higher abundance (Yu et al., 2019). This assertion is confirmed in the present study wherein, in the 40% HA-starch diet, we observed a reduced abundance of *Streptococcus,* possibly because of the competitive exclusion by *Lactobacillus.* The 2 most abundant *Streptococcus* species are *S. hyointestinalis* and *S. gallolyticus* (Devriese et al., 1988; Dumke et al., 2015; Pasquereau-Kotula et al., 2018a). The addition of 40%HA-starch increased the abundance of *S. hyointestinalis,* whereas the addition of 40%CPCC increased the abundance of *S. gallolyticus*. *S. hyointestinalis* was reported to produce antimicrobial protein molecules with unique characteristics similar to bacteriocins (Lee et al., 2020). *S. gallolyticus* reportedly belongs to a few opportunistic human pathogens (Pasquereau-Kotula et al., 2018b). Exploring fecal microbiota composition and metagenomic functions associated with feed efficiency in pigs as reported by Quan et al. (2019) suggested that sub-species *S. gallolyticus* subsp. *gallolyticus* could be a key candidate for improving feed efficiency. Our findings show that the relative abundance of *Terrisporobacter* genera increased in pigs fed a diet containing 40%HA-starch. Similarly, dietary protein restrictions (low-protein diet) in piglets increased the proportion of *Terrisporobacter* abundance (Liu and Fan, 2023). The consumption of basal diet with 50 g/kg wheat bran and 50 g/kg fermented wheat bran increased the abundance of *Terrisporobacter* to as high as 47% in pigs (He et al., 2023).

The diversity and richness of the cecal microbiome are pivotal factors in both the digestion of dietary nutrients and the fermentation of indigestible starch within the hindgut. Alterations in dietary composition exert a notable influence on microbial diversity and richness. In the present investigation, a substantial augmentation in microbial diversity and richness was noted in piglets-fed diets containing either 40%CPCC or 40%HA-starch. Similarly, Tachon et al. (Tachon et al., 2013) observed microbial diversity and richness escalation upon introducing type 2 resistant starch from high-amylose maize to mice. Conversely, Tiwari et al. (Tiwari et al., 2022) found no significant shifts in bacterial diversity and composition when administering starchy and fibrous feed to pigs. Furthermore, a previous report by Metzler-Zebeli et al. (2015) indicated no noteworthy alterations in cecal bacterial diversity and richness when resistant starch type 4 was provided to growing pigs. The dissimilarity in observations between this study and previous research could stem from differences in pig selection and the composition of high-amylose sources. We employed weaned piglets in our study, whereas the previously reported studies used growing-finishing pigs. The presence of HA-starch impacted both the alpha diversity of the cecal microbiome and the overall abundance of bacterial 16S rRNA genes in the cecal digesta. The Venn diagram clearly distinguished that the HA-starch diet supported a greater diversity of bacteria in the cecal digesta than the control diet. Additionally, this effect was observed in comparison to diets supplemented with CPCC. Notably, the cecal digesta from the diet enriched with HA-starch exhibited more distinct bacterial taxa than the control diet and the diets supplemented with CPCC. Similarly, Metzler-Zebeli et al. (2015) reported an increase in unique cecal bacterial taxa in pigs fed on enzymatically modified starch. The microbiota composition of piglets fed 40%HA-starch with no CPCC (40%HA-starch + 0%CPCC) had a significantly distinct bacterial composition, clustered separately from the other treatment groups with variation due to dietary inclusion of HA-starch. This is evident with the differential enrichment of cecal bacterial genera such as *Enterorhabdus*, *Eggerthellaceae_unclassified*, *formicigenerans*, *ponci*, *pontis*, *Terrisporobacter*, and *amylovorus*. Similarly, resistant starch consumption modulated the compositional structures of human intestinal microbiota (Martínez et al., 2010) and the cecal microbiota of swine (Haenen et al., 2013; Umu et al., 2015).

In summary, incorporating HA-starch and CPCC into the diet of weaned piglets led to the modulation of the intestinal microbiota. This modulation was characterized by an augmentation in microbial diversity and an increased abundance of probiotic commensal bacteria. Specifically, diets enriched with HA-starch increased the relative abundance of the Lactobacillus and Terrisporobacter genera. Notably, the introduction of HA-starch in the piglet diet correlated with increased *S. hyointestinalis*, while piglets fed a CPCC diet exhibited higher proportions of *S. gallolyticus*. This enhancement in probiotic strains contributed to heightened enzyme activity involved in substrate degradation, consequently leading to an increase in the production of VFAs, which serve as a significant energy source for microbial communities and host organisms.

## Supplementary Material

skaf008_suppl_Supplementary_Materials

## Abbreviations

ASV: amplicon sequence variants
BCFA: branched-chained fatty acid
CPCC: Cold-pressed canola cake
HA-starch: high-amylose cornstarch
PERMANOVA: permutational multivariate analysis of variance
SBM: soybean meal
VFA: volatile fatty acid
