# Supplementary material for: Dietary inclusion of high-amylose cornstarch increased Lactobacillus and Terrisporobacter and decreased Streptococcus in the cecal digesta of weanling pigs
Source: J Anim Sci. 2025 Jan 18;103:skaf008. doi: 10.1093/jas/skaf008 (PMC12056938; doi:10.1093/jas/skaf008)
Supplement: skaf008_suppl_Supplementary_Materials [file skaf008_suppl_supplementary_materials.docx]

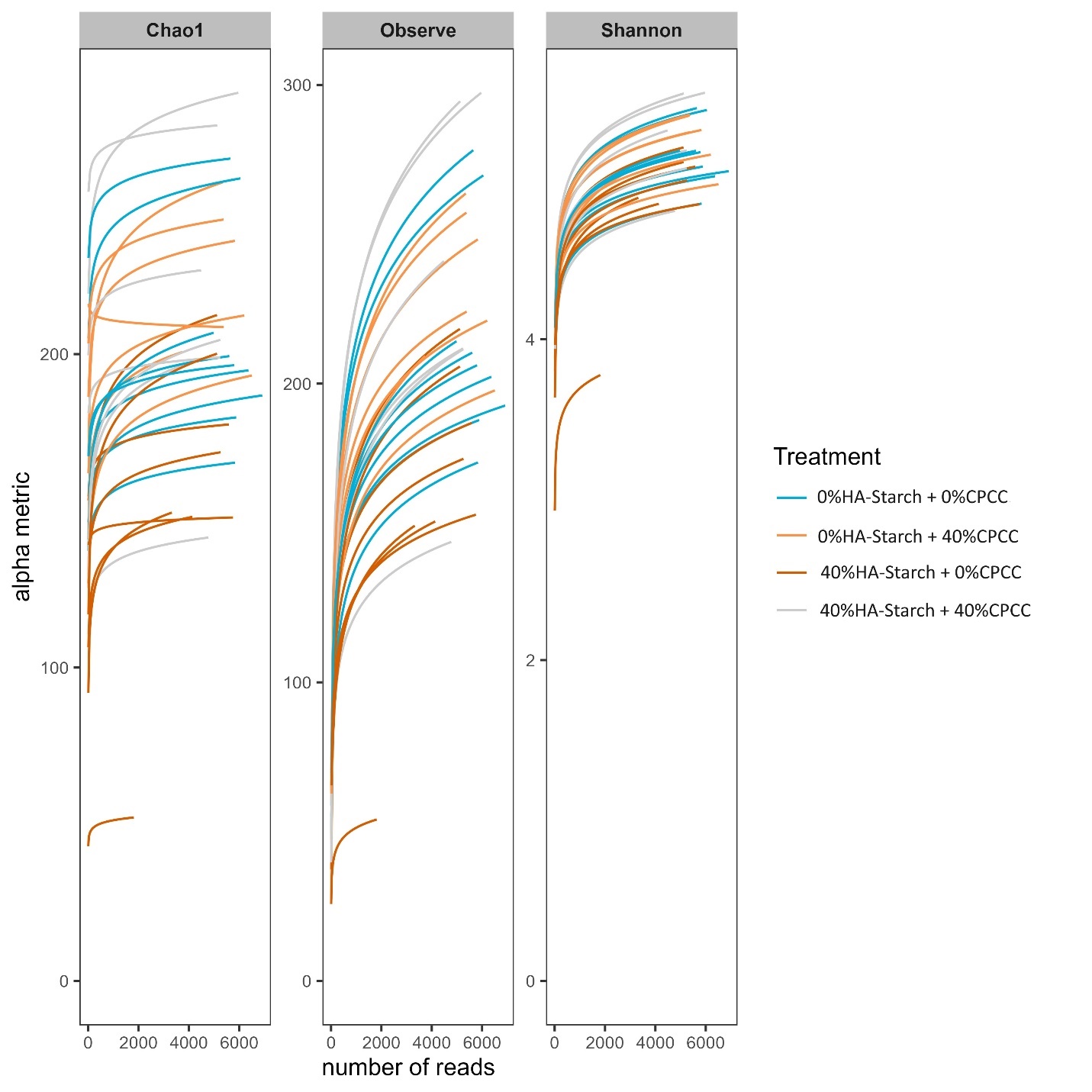


**Supplementary Fig 1:** Rarefied read abundance based on alpha diversity matrices**.**


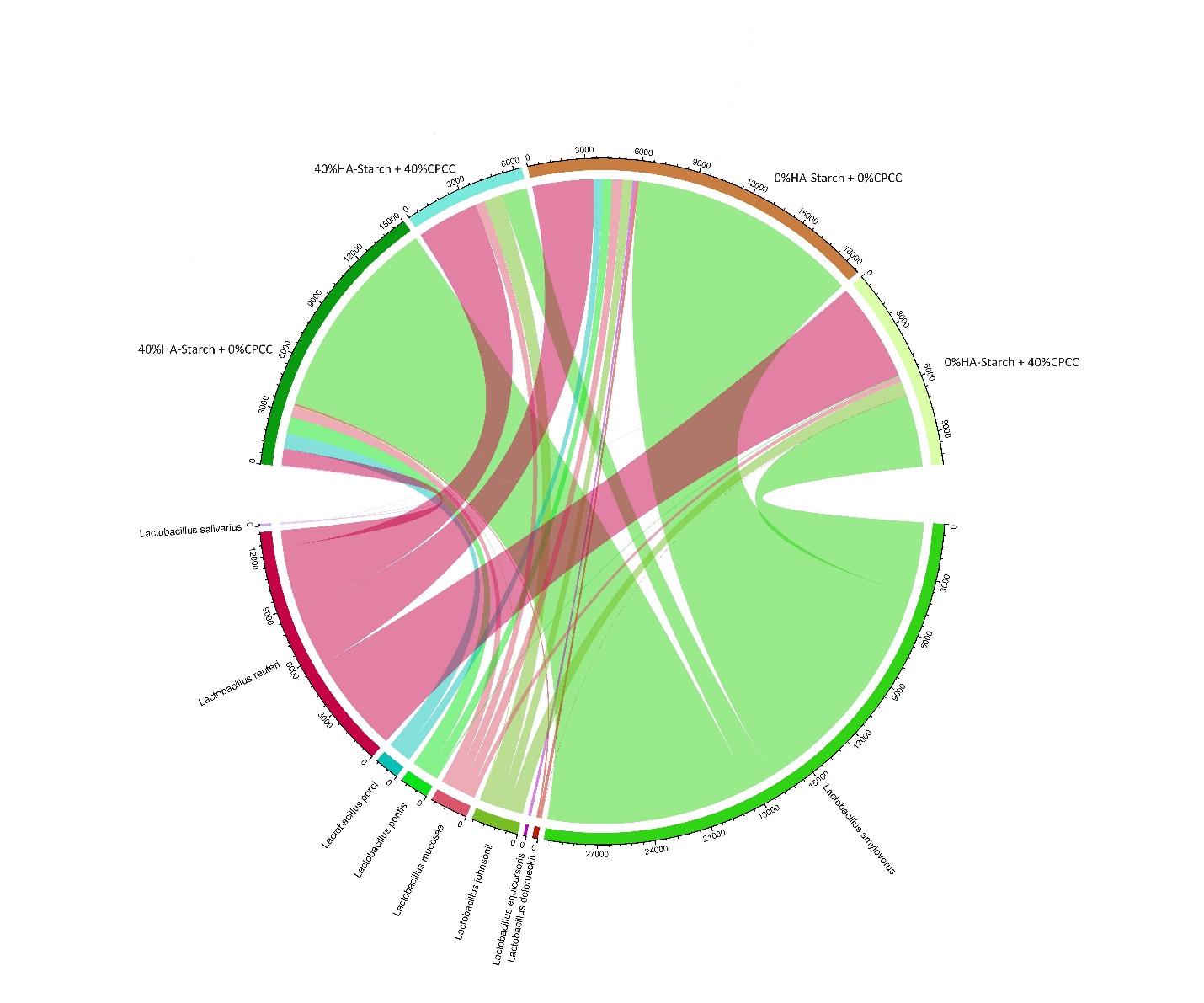


**Supplementary Fig 2.** Circos plot of total read abundance of Lactobacillus species in the treatment group.
